# Lessons from developing the Papua New Guinea National Health Research Agenda 2025–2030

**DOI:** 10.1016/j.lanwpc.2026.101830

**Published:** 2026-03-17

**Authors:** Manah Dindi, Greco B. Malijan, Gui Xian Ong, Paik Tade, Peter Siba, Natasha Cooke, Mengji Chen, Kidong Park, Priya Mannava

**Affiliations:** a Performance, Monitoring and Research Branch, National Department of Health, Port Moresby, Papua New Guinea; b Division of Data, Strategy and Innovation, WHO Regional Office for the Western Pacific, Manila, Philippines; c Medical Research Advisory Committee, National Department of Health, Port Moresby, Papua New Guinea; d Centre for Health Research and Diagnostics, Divine World University, Madang, Papua New Guinea; e Division of Healthy Environments and Populations, WHO Regional Office for the Western Pacific, Manila, Philippines; f WHO Country Office in Papua New Guinea, Port Moresby, Papua New Guinea

Papua New Guinea's (PNG) National Health Plan (NHP) 2021–2030 articulates a vision of “a healthy and prosperous nation where health and wellbeing are enjoyed by all,” anchored in evidence-based innovation as a fundamental value.^1^ To operationalise this vision and support evidence generation to address the country's complex health challenges, the National Department of Health coordinated the development of the “National Health Research Agenda (NHRA) 2025–2030” in 2024–2025. The NHRA is envisioned to serve as a strategic guide for health research system actors and institutions to align health research efforts and investments with local priorities. The development of NHRA 2025–2030 builds upon the lessons learned from the prior research priority-setting exercise in PNG in 2012, which led to the development of the first Health Research Policy.^2^

Guided by the WHO health research prioritisation framework,^3^ the NHRA development methodology combined systematic evidence review, inclusive stakeholder engagement, foresight analysis, and inductive reasoning to ensure research priorities directly support NHP implementation. Initial scoping interviews ensured representation across research, policy, and implementation perspectives. A health research situation analysis mapped studies published between 2020 and 2024 across six domains (communicable and non-communicable diseases, family health, healthy lifestyles, health systems, and health security), while national health indicators^4^^,^^5^ were analysed to assess alignment with research conducted in PNG.

A two-stage consultation process generated and refined research priorities: Stage 1 (November 2024) used foresight analysis,^6^ gap reviews, and nominal group technique to foster discussions across interest-holders, generating 62 research questions; Stage 2 (May 2025) refined these into research statements prioritised against feasibility, impact, and ethical criteria, with the priority setters also identifying governance, funding, and capacity-building actions for implementation.

## Strategic priorities with implementation focus

The agenda encompasses 57 research statements, organised into domains and by implementation timeframes (Table 1). The health systems domain had the greatest number of statements, reflecting the foundational role of systems strengthening for improved outcomes across all health programs.

**Table 1:** Summary of priority research statements from the PNG NHRA 2025–2030.

Timeframe^a^	Research topic	Research statement
Domain 1: communicable diseases		
Short-term	Tuberculosis & leprosy	Factors influencing treatment adherence and follow-up among individuals who default on TB treatment
	Malaria, vector-borne diseases and NTDs	Evaluating the effectiveness of solutions for *Plasmodium falciparum* and *Plasmodium vivax* malaria, including arbovirus component, using widespread local involvement
Medium-term	HIV, STIs & hepatitis	Identify solutions to attendance to health care services and adherence to treatment of TB, HIV and other STIs
	Communicable diseases	Investigation into implementation strategies that enhance coverage and uptake of treatments for communicable diseases
		Prevalence of communicable diseases (TB, HIV, STIs and NTDs) among the broader populations in priority provinces in PNG
		Mapping opportunities for multi-disease screening (for NCDs and CDs) among at risk populations
	Malaria, vector-borne diseases and NTDs	Impact of climate change (and environmental changes) on host and vector behaviours, genetics and vector-borne disease prevalence
		Determinants influencing *Plasmodium falciparum* and *Plasmodium vivax* malaria rates in PNG population
	Tuberculosis & leprosy	Mapping the resources and training needs for healthcare workers and other community actors to enable multi-disease screening and address the high TB burden among at risk population, including DMT2
		Cost-benefit of various TB interventions (including drug-resistant TB regimens)
		Enablers and barriers to addressing stigma within the clinical control efforts towards TB, leprosy and other skin-NTDs among community and service providers
Long-term	Communicable diseases	Investigation into impacts of Papua New Guinean cultural practices and social norms on infectious disease transmission
Domain 2: non-communicable diseases		
Medium-term	Cancer	Investigation into patient management, and efficacy of cancer treatments and potential causes of treatment resistance
		Investigation into prevalence and incidence of different cancer types within the PNG population
	Disabilities	Assess health services for inclusion & accessibility for individuals with disability
		Characterisation (on types & prevalence) of disabled persons in PNG
	Non-communicable diseases	Knowledge and attitudes towards NCDs in PNG (adapted to specific populations)
	Injuries & violence	Social and political determinants of and solutions for ethnic violence across PNG
		Quantify the burden of trauma and access to care for injury and trauma
Domain 3: health lifestyles		
Short-term	Health promotion	Effectiveness of various communication tools and public health messages on healthy lifestyles in PNG
Medium-term	Mental health	Estimate the prevalence of mental health disorders in PNG
	Oral health	Assess the status of oral health in PNG
	Alcohol & substance abuse	Examination of the determinants of rising drug and substance abuse among general and youth populations
Domain 4: family health		
Short-term	Adolescent health	Review of available data on adolescent health in PNG and identify gaps and priority areas for adolescent health
Medium-term	Gender & violence	Identify multi-disciplinary solutions and connect interdisciplinary services for violence against women & children
		Assessment of methods to engage men in issues of men's health including violence against men
		Evaluate fragmentation of measurement of violence against women & children to identify gaps
	Maternal & newborn health	Identify factors influencing declines in utilising essential reproductive and child health services and potential solutions to improve utilisation
	Adolescent health	Identification of optimal delivery of healthcare to adolescents in PNG
		Identify healthcare interventions for adolescents through schools and communities
	Reproductive health	Identify solutions to address the determinants of use and gaps in reproductive and family planning services for young couples
Domain 5: health system		
Short-term	Leadership, governance, laws & legislation	Evaluation of health research translation into policy and implementation over the last 5 years
	Service planning & delivery	Determine the factors associated with health infrastructure and service gaps for communicable diseases
		Explore support mechanisms to strengthen PHA capacity for healthcare service delivery
	Health workforce	Assessment of PNG health worker capacity to respond to health emergencies
	Health financing	Explore optimal approaches to increase investments from traditional and non-traditional donors
Medium-term	Leadership, governance, laws & legislation	Evaluate strategies that contribute to strengthened health system governance and multi-sector partnerships for improved health outcomes
	Surveillance, M&E & health information	Evaluate the performance of eNHIS over the past 5 years to improve or strengthen electronic health information system across all health programs.
	Essential medicines and technologies	Evaluation of the current logistics management information system in improving accessibility and availability of essential medicines
	Digital health & innovation	Assessment of digital health literacy skill levels across health facilities in PNG
	Health financing	Investigation into optimal financial model(s) for provincial health authorities to distribute funding
		Evaluation of return on healthcare investments on health systems strengthening and its impact for health system coverage
	Service planning & delivery	Explore options for establishing public health teams in health facilities
		Investigation of high-risk populations' health-seeking behaviors and treatment adherence for high-burden diseases
		Investigation of factors leading to low utilisation of health services and measures that can improve utilisation at the national and sub-national levels in PNG (disaggregated by types of services and population subgroups)
		Evaluation of health information product and strategy utilisation to improve service delivery of PHAs
	Primary healthcare	Impact of free primary healthcare policy on health system performance
		Strategies to improve the PHC model for specific provinces to reach rural areas with far and hard-to-reach populations
	Health workforce	Investigation into measures which improve production, registration, retention, compliance and exit of health professional practitioners
Domain 6: health security		
Medium-term	One Health	Determine the factors enabling multi sectoral collaboration in One Health in PNG
	Antimicrobial resistance	Determine the factors associated with non-adherence to antibiotic use
		Investigation of the burden of anti-microbial resistance in PNG, both in disease and economic burden
		Investigation into the scale of out-of-health-system supply chain of basic antibiotics (e.g. how many people are using the antibiotics)
	Zoonotic diseases	Estimate the prevalence of zoonotic diseases in PNG
	Health emergencies & outbreaks	Cost-benefit evaluations of interventions and programs for pandemic preparedness from a societal perspective in PNG
		Evaluation of PHA implementation approaches for effective emergency response
Long-term	Climate risks, climate change resilience & preparedness	Impact of climate change on health systems & health security, including food security and environmental health

a Short-term—research that can be completed within 1–2 years, Medium-term—research requiring 3–5 years to generate comprehensive evidence, Long-term—research that may commence within the next 5 years but is likely to produce evidence over a longer timeframe.

Acknowledging the importance of translating high-level agendas to action, the NHRA includes an implementation framework often overlooked from priority-setting exercises: (1) strengthening governance with harmonised ethics processes and streamlined oversight and the Medical Research Advisory Committee serving as the central, coordinating body; (2) sustainable funding through a pooled, competitive domestic health research fund and a piloted grant mechanism; (3) capacity building across methods, data, and knowledge translation including curricula reform and proposal-writing support; and (4) monitoring, evaluation, and adaptive learning with a set of indicators that track local leadership, domestic funding share, and mixed-methods and interdisciplinary uptake.

## Lessons for regional application

### Co-designing the NHRA development methodology improves feasibility and ownership of the priority setting process

Meaningful stakeholder engagement preceded any draft list, with early interviews across central and provincial health authorities and research institutions identifying opportunities that can be leveraged from previous prioritisation exercises, shaping the scope of current agenda setting, and providing the vision for how the NHRA could meaningfully contribute to improving health outcomes. Furthermore, this step surfaced bottlenecks that would later be addressed in the implementation framework. It also improved legitimacy of the procedures, set expectations, and anchored priorities to NHP delivery needs.

### Systematic health research output mapping can offer useful insights beyond research priorities

Mapping recent publications in PNG provided additional evidence on who leads, funds, and oversees health research in the country, driving meaningful discussions on addressing challenges related to local research ownership and funding. Sharing these findings with the stakeholders prior to any research prioritisation added depth to developing research priorities, expanding considerations for initiatives that could expand local research capacities.

### The choice of methods and prioritisation criteria is an ethical exercise, influencing whose needs are addressed first and how resources are allocated

Nominal group technique was selected to provide a structured process (involving a silent brainstorming, round robin, and a moderated group discussion), supporting equal contribution and reducing the risk that a few participants disproportionately shape the discussion. A scoring matrix was applied to prioritise the statements systematically against the criteria developed and agreed prior to scoring. The criteria were co-developed by the priority-setting participants, comprised of representatives from varying National Department of Health programme areas, the Medical Research Advisory Committee, provincial health authorities, the church health sector, and local universities and research institutions. The breadth of sectoral expertise and perspectives across this group provided insight into both anticipated public health impact and practical considerations of feasibility and contextual fit. Making these choices transparent, and ensuring they reflect fairness, inclusivity, and local realities, are essential for to agenda to be both legitimate and impactful.

### Bringing implementers and funders into the room enables pragmatic pathways to action

Purposeful inclusion of research institutions, central agencies, development partners, and potential funders alongside health programme managers built a shared vision and highlighted alignment opportunities beyond the health sector, and increased the odds that priorities become funded, locally led studies. This approach is envisioned to increase the likelihood that the set priorities can be translated into funded, locally-led studies. Domestic ownership and investment could be secured by early engagement of central agencies during implementation.

### Priority lists benefit from consideration of timelines and a learning system

Considering evidence-generation horizons helped test present assumptions while anticipating future needs. While strategic foresight is usually conducted as a separate exercise, integrating it into this process supports creating a more cohesive and forward-looking implementation and evaluation approach focused on uptake, equity, capacity gains, and systems change.

PNG's experience suggests that treating research priority-setting as a system intervention, not a catalogue of topics, yields more value. Three transferable features to similarly resourced settings and other Pacific Island countries include: (i) early co-design with stakeholders; (ii) systematic evidence review; and (iii) designing implementation strategy alongside the priorities. Framed this way, a national health research agenda can become a strategic tool for system transformation, shifting research activity from ad-hoc projects to coordinated, accountable programmes linked to national health plans and addressing local health needs.

Realising this potential requires coordinated implementation across government, academia, health providers, communities, and development partners. Sustained domestic resourcing remains the principal uncertainty. The proposal for phased allocations to a national research fund, pooled with industry and partner contributions and awarded competitively, offers a pragmatic starting point. Ongoing stakeholder involvement and transparent governance will be essential to make these mechanisms durable and to keep the agenda anchored in local leadership and public health impact. Sustained collaboration with agencies beyond the health sector can help advance the allocation of resources towards NHRA implementation despite financial constraints.

## Conclusion

The PNG NHRA 2025–2030 reframes health research in the country as a shared enterprise: priorities with timelines, governance that can act, and financing and capacity measures that enable local leadership. Delivering on this agenda will require coordinated effort by the community of practice including central and provincial government, academia, communities, clinicians and public health practitioner, funders and development partners. If implemented as designed, the agenda can accelerate evidence generation that can inform health policy to improve health outcomes in PNG. These methods offer a practical template for countries seeking to align health research and investments with the greatest health needs in resource-constrained settings.

## Declaration of interests

None.
